# Metformin suppresses proliferation and differentiation induced by BMP9 *via* AMPK signaling in human fetal lung fibroblast-1

**DOI:** 10.3389/fphar.2022.984730

**Published:** 2022-08-24

**Authors:** Qiongfeng Chen, Yaqun Wang, Linna Sheng, Yonghong Huang

**Affiliations:** ^1^ Department of Pathophysiology, Basic Medical College of Nanchang University, Nanchang, China; ^2^ Department of Pathology, Basic Medical College of Nanchang University, Nanchang, China

**Keywords:** metformin, AMPK, BMP9, lung fibroblast, proliferation, differentiation

## Abstract

Adenosine monophosphosphate-activated protein kinase (AMPK) and its activator metformin were found to be involved in the regulation of fibroblast activation and pulmonary fibrosis. However, the regulatory mechanism has been undetermined. Recently, AMPK has been reported to exert its effect through inhibiting bone morphogenetic protein (BMP) pathway. In this study, human fetal lung fibroblast (HFL-1) cells were treated with metformin or specific AMPKα1 mutants, including constitutively activated mutant (AMPK-CA) and dominant negative mutant (AMPK-DN), combined with BMP9, and then the absorbance of these cells was measured by cell counting kit (CCK)-8 assay. The colony number of HFL-1 cells stimulated by metformin with or without BMP9 was examined by colony formation assay. The protein expressions of differentiated markers (α-smooth muscle actin, collagen I and collagen III) and the key molecules of BMP9 signaling, including activin receptor-like kinase (ALK) one and phosphorylated small mother against decapentaplegic (p-Smad)1/5, were also evaluated by western blot. Data revealed that BMP9 induced the proliferation and differentiation of HFL-1 cells which was suppressed by metformin or AMPK-CA. Meanwhile, the effect of metformin on BMP9-induced activation was counteracted by AMPK-DN. In addition, we found that the expressions of ALK1 and p-Smad1/5 induced by BMP9 were attenuated by metformin and AMPK-CA, whereas the inhibitory responses of metformin to the increased ALK1 and p-Smad1/5 were reduced by AMPK-DN. Accordingly, these results suggested that metformin mitigated BMP9-induced proliferation and differentiation of HFL-1 cells, which was achieved partly through the activation of AMPK and inhibition of ALK1/Smad1/5 signaling.

## Introduction

Pulmonary fibrosis is a kind of chronic lung disease with poor prognosis, and is also one of the refractory diseases in respiratory system (Noble et al., 2012; Hutchinson et al., 2015). The underlying reason for the poor treatment might be that the pathogenesis of pulmonary fibrosis has not been fully elucidated. Increasing studies have indicated that lung fibroblasts are the master participants in the development of pulmonary fibrosis (Crapo et al., 1982; Andersson-Sjöland et al., 2008). Their abnormal activation, including excessive proliferation and transdifferentiation into myofibroblasts, can result in the accumulation of extracellular matrix, and eventually lead to pulmonary fibrosis (Gabbiani et al., 1976; Darby et al., 2016; Hinz, 2016). Consequently, the intervention of fibroblast activation can be a crucial pathway to attenuate pulmonary fibrosis.

Metformin is a widely used biguanide antidiabetic medication in the patients with type 2 diabetes (Sanchez-Rangel and Inzucchi, 2017), and is also an important activator of adenosine monophosphosphate-activated protein kinase (AMPK) (Foretz et al., 2014; López, 2018; Chen et al., 2020). In animal experiments, metformin was reported to not only mitigate bleomycin- and radiation-induced lung injury and pulmonary fibrosis through the activation of AMPK (Choi et al., 2016; Azmoonfar et al., 2018; Farhood et al., 2019; Xiao et al., 2020), but also protect against fine particulate matter (PM_2.5_, aerodynamic diameter≤ 2.5 μm)-induced lung injury and fibrosis through AMPK independent pathway (Gao et al., 2020). In cellular experiments, studies found that metformin-mediated activation of AMPK inhibited transforming growth factor (TGF)-*β*-induced proliferation, differentiation and collagen production of lung fibroblasts (Wang et al., 2020). Mechanistically, some research indicated that metformin prevented fibroblasts activation and lung fibrosis through suppressing the activity of nicotinamide adenine dinucleotide phosphate of oxidase 4 (NOX4) and oxidative stress (Jarman et al., 2014; Sato et al., 2016), whereas other research found that several different factors, such as TGF-β (Li et al., 2015), transglutaminase 2 (Wang et al., 2020), insulin-like growth factor (IGF)-1 (Xiao et al., 2020), bone morphogenetic protein (BMP)2 (Kheirollahi et al., 2019), and mammalian target of rapamicin (mTOR) (Shao et al., 2015; Rangarajan et al., 2018), were involved in the amelioration of lung fibrosis mediated by AMPK and its activator metformin. It follows that the mechanism of metformin against pulmonary fibrosis is rather complex. Whether there is an additional mechanism during the process of metformin-mediated anti-fibrosis in the lung has been undetermined.

Recent studies reported that AMPK inhibited the BMP signaling pathway to play its biological function. For example, metformin not only inhibited BMP6-induced osteoblast differentiation through type I BMP receptor activin receptor-like kinase (ALK) two and its downstream transcriptional factor small mother against decapentaplegic (Smad) 1/5 (Lin et al., 2017), but also suppressed the tube formation of human umbilical vein endothelial cells (HUVECs) induced by BMP9 via ALK1/Smad1/5 signal pathway (Ying et al., 2017). Lately, BMP9 was shown to induce the proliferation and elevate the expression of *α*-smooth muscle actin (α-SMA) and collagen proteins in hepatic stellate cells, and thereby promote hepatic fibrosis (Li, Zhao et al., 2008; Bi and Ge, 2014; Breitkopf-Heinlein et al., 2017; Li et al., 2018). Furthermore, our latest study demonstrated that BMP9 activated human fetal lung fibroblast HFL-1cells *in vitro* (Wang et al., 2021). In this study, we further interrogated the effect of metformin on HFL-1 cells treated with BMP9, as well as the relationship between AMPK and BMP9 pathway in the *in vitro* model, to elucidate the mechanism of metformin-mediated anti-fibrosis.

## Materials and methods

### Cell culture

Human fetal lung fibroblast (HFL-1) cells (Shanghai Cell Bank of Chinese Academy of Sciences) were cultured in dulbecco’s modified Eagle’s medium (DMEM, HyClone; Cytiva) containing 6% fetal bovine serum (FBS, HyClone, Cytiva) and 1% penicillin/streptomycin (Solarbio, Beijing, China) in the conventional culture condition (5% CO_2_, 37°C and saturated humidity). Cells used in these experiments were within cell passges 5 after cell recovery. All HFL-1 cells get starved for 6 h before treatment.

### Cell viability assay

Cell viability assay was performed as described previously (Wang et al., 2021). In a word, cell viability was detected by Cell Counting Kit-8 (CCK-8, Transgen, Beijing, China) assay to evaluate the proliferation of HFL-1 cells according to the manufacturer’s data sheets. Based on the requirements of different treatments, HFL-1 cells were divided into different groups. In each group, DMEM (100 μl) containing 1,000 cells was added to each well in a 96-well plate and incubated for the corresponding time at 37°C, followed by the addition of 10 μl CCK-8 reagent. The absorbance of each well was then detected at a wavelength of 450 nm by a multi-mode microplate reader (Molecular Devices, United States). Cell proliferation was represented as the relative absorbance.

### Colony formation assay

The assay was carried out as described previously (Wang et al., 2021). Briefly, in each group, 1,000 cells were evenly spread in a 6-cm dish and cultured continuously for 10 days. During the process, the culture medium was replaced every 3 days. After incubation, the cells in each group were gently washed with phosphate buffer solution for 2 times, and fixed with 4% paraformaldehyde at room temperature for 20 min, followed by rinsing with phosphate buffer solution. And 1% crystal violet solution (Solarbio, Beijing, China) was then added for staining at room temperature for 15 min. The images of the forming colonies in the 6-cm dish were collected by Scanner, and then the colony number was counted by ImageJ software (NIH, United States).

### Adenovirus preparation and infection

The recombinant plasmids, including constitutive activation mutant of AMPKα1 (AMPK-CA), dominant negative mutant of AMPKα1 (AMPK-DN) and adenovirus vector with green fluorescent protein (GFP-AD), were the gifts from Dr. Zhijun Luo of Nanchang University. These plasmids were successfully tested in his research (Lin et al., 2015; Ying et al., 2017). 70% of HFL-1 cells were plated in a 6-cm dish and cultured overnight in DMEM medium with 6% FBS, and then replaced 0.1% FBS for 6 h at 37°C. Subsequently, adenovirus-containing FBS-free DMEM medium displace them for 4 h at 37°C. After this, the cells were continuously cultured in 6% FBS medium for 48 h with or without drug treatment.

### Western blot

Western blot was performed according to the standard protocal. Briefly, in each group, cells (5 × 10^6^) in a 6-cm dish were lysed by RIPA lysis buffer (Beyotime, Shanghai, China). Total protein were collected after centrifugation and quantified by a Bicinchoninic Acid Protein Assay Kit (Beyotime, Shanghai, China). Protein samples (20 or 40 μg) were separated with 8% or 10% sodium dodecyl sulfate polyacrylamide gel electrophoresis and transferred onto nitrocellulose membranes (Millipore, United States). The membranes were blocked in 5% non-fat dry milk at room temperature for 1 h, and then incubated overnight at 4°C with specific primary antibodies against total (t)-Smad1/5, phosphorylated (p)-Smad1/5, p-AMPK, AMPK (1:1,000, Cell signaling Technology, United States), Collagen I, Collagen III, ALK1 (1:1,000, Affinity Biosciences, China), ɑ-SMA (1:10,000, Abcam,United States) and glyceraldehyde phosphate dehydrogenase (GAPDH, 1:5000, Affinity Biosciences, China). The next day, the membranes were incubated with the corresponding horseradish peroxidase-conjugated secondary antibodies (1:5000, Affinity Biosciences, China) at room temperature for 2 h. Protein bands were visualized with enhanced chemiluminescence (Milliprore, United States) according to the manufacturer’s protocol. The relative protein expression was quantified using ImageJ software (NIH, United States) and normalized with GAPDH.

### Statistical analysis

All data were analyzed by SPSS 17.0 statistical software. The measurement data were expressed by mean ± standard deviation (SD) from at least three independent experiments. Comparisons among multiple groups were analyzed by one-way ANOVA followed by Tukey’s post hoc test. *p* < 0.05 was considered statistically significant.

## Results

### Metformin inhibited BMP9-induced proliferation and differentiation of HFL-1 cells

**FIGURE 1:**
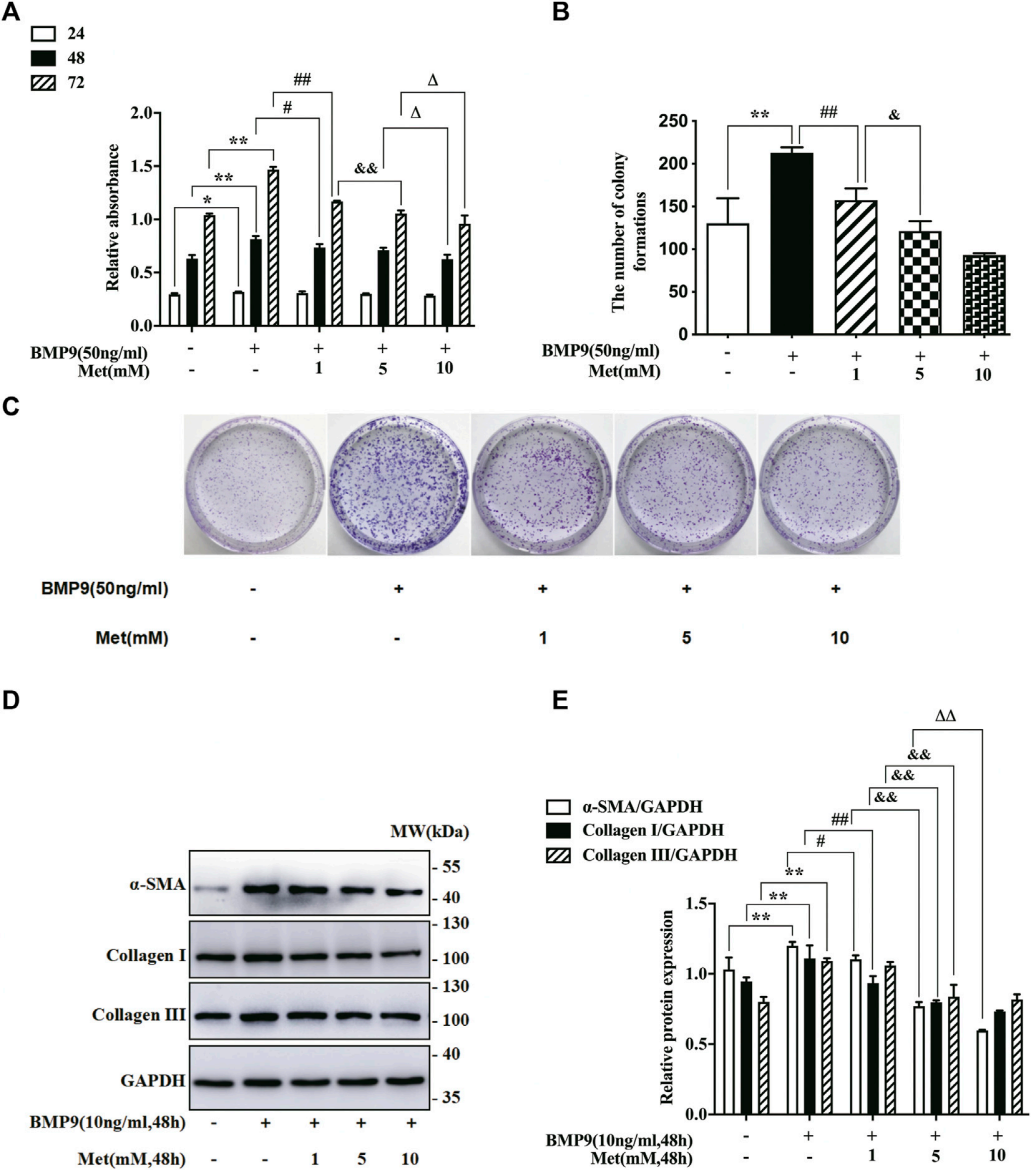
Metformin inhibited BMP9-induced proliferation and differentiation of HFL-1 cells. **(A)**: HFL-1 cells were treated with different concentrations of metformin (0, 1, 5 and 10 mM) combined with BMP9 (50 ng/ml) for 24, 48 and 72 h. The proliferative ability was detected using a CCK-8 assay and represented as the relative absorbance (*n* = 3). **(B,C)**: HFL-1 cells were treated with different concentrations of metformin (0, 1, 5 and 10 mM) combined with BMP9 (50 ng/ml) for 10 days. The ability of colony formation was evaluated using colony formation assay (*n* = 3). **(D,E)**: HFL-1 cells were treated with different concentrations of metformin (0, 1, 5 and 10 mM) combined with BMP9 (10 ng/ml) for 48 h. The expression levels of fibroblast differentiation marker proteins (ɑ-SMA, Collagen I, Collagen III) were examined by western blot (*n* = 3). Cells dealt with only culture medium were served as a control group. Data are presented as mean ± SD. ^*^ *p* < 0.05 and ^**^ *p* < 0.01 versus control group; ^#^ *p* < 0.05 and ^##^ *p* < 0.01 versus only BMP9 stimulated group; ^&^ *p* < 0.05 and ^&&^ *p* < 0.01 versus the group exposed to 1 mM of metformin with BMP9; ^Δ^P < 0.05 and ^ΔΔ^P < 0.01 versus the group exposed to 5 mM of metformin with BMP9.

Increased proliferation and differentiation are the essential properties of activated fibroblasts (Wynn and Ramalingam, 2012). Thus, to determine whether metformin affected the activation of HFL-1 cells, we explored the role of metformin in BMP9-stimulated HFL-1 cells. Firstly, we used CCK-8 and colony formation assay to examine the proliferation of HFL-1cells exposed to different concentrations of metformin (0, 1, 5 and 10 mM) combined with BMP9 (50 ng/ml) at the indicated time (24, 48 and 72 h). The cells only with culture medium were served as a control group. The CCK-8 assays showed that BMP9 significantly increased the relative absorbance, which was restrained by metformin in a concentration-dependent manner at the time of 72 h (Figure 1A). Consistent with CCK-8 assays, the results of colony formation assays also confirmed that BMP9 strongly enhanced the colony number, whereas metformin concentration-dependently reduced the BMP9-induced colony formation (Figures 1B,C). Next, western blot was detected the protein expression levels of fibroblast differentiation markers (ɑ-SMA, Collagen I and Collagen III) in the HFL-1 cells treated with different concentrations of metformin (0, 1, 5 and 10 mM) combined with BMP9 (10 ng/ml) for 48 h. As shown in Figures 1D,E, the protein expressions of ɑ-SMA, Collagen I and Collagen III were up-regulated after BMP9 treatment, while metformin reduced their up-regulations. Therefore, these data indicate that BMP9 can induce the proliferation and differentiation of HFL-1 cells, which can be suppressed by metformin.

### Role of metformin in the expression of BMP9 downstream signaling molecules and AMPK

**FIGURE 2:**
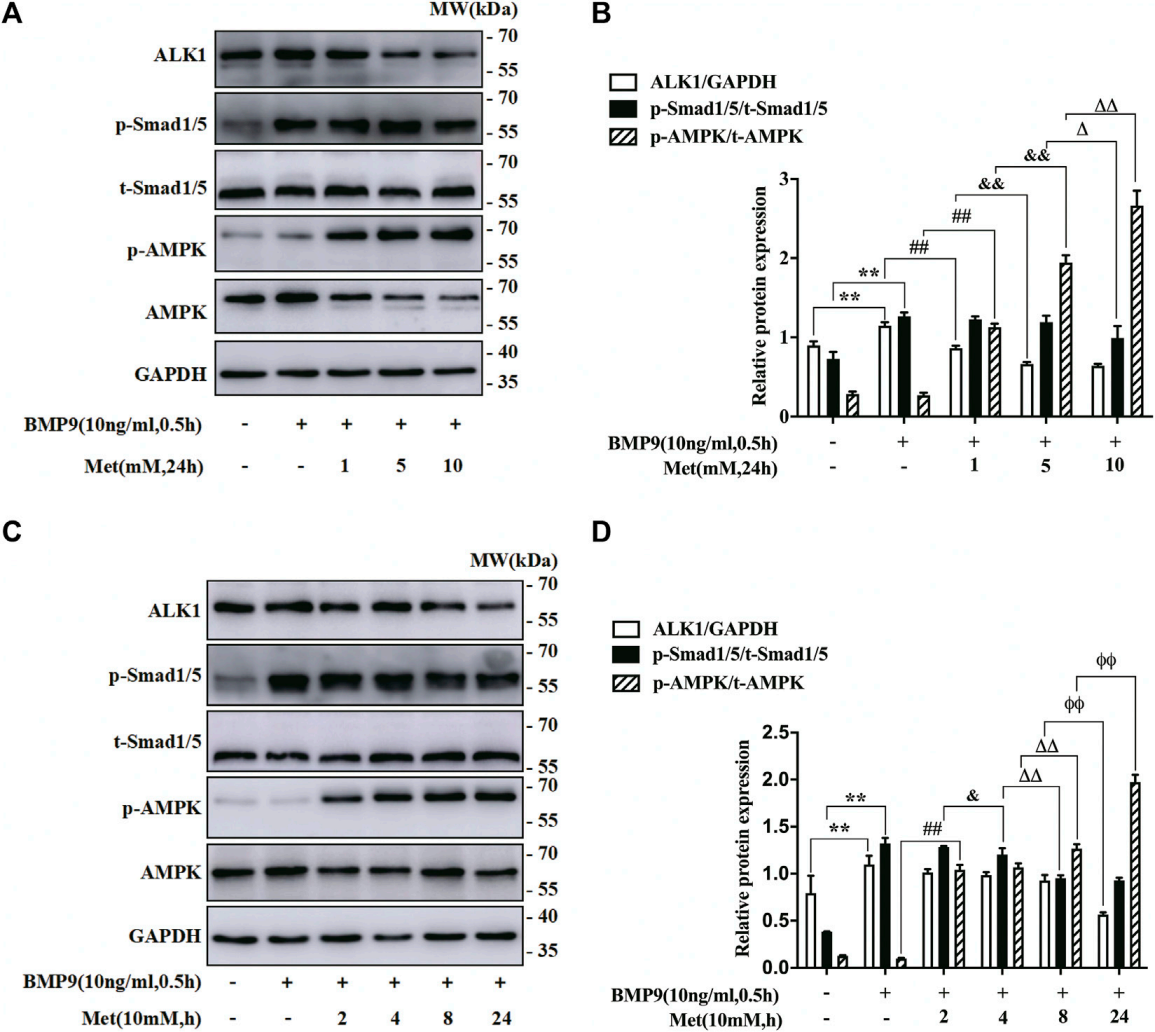
Role of metformin in the expression of BMP9 downstream signalling molecules and AMPK. **(A,B)**: Western blot was used to detect the expression of p-AMPK, ALK1 and p-Smad1/5 in HFL-1 cells with different concentrations of metformin (0, 1, 5, 10 mM) and 10 ng/ml BMP9 for 0.5 h (*n* = 3). **(C,D)**: Western blot was applied to test the expression of p-AMPK, ALK1 and p-Smad1/5 in HFL-1 cells with 10 mM metformin for different times (2, 4, 8 and 24 h) and 10 ng/ml BMP9 for 0.5 h. Cells dealt with culture medium were served as control group (*n* = 3). Data are presented as mean ± SD. ^**^ *p* < 0.01 versus control group; ^##^ *p* < 0.01 versus only BMP9 stimulated group; ^&^ *p* < 0.05 and ^&&^ *p* < 0.01 versus 1 mM metformin for 24 h or 10 mM metformin for 2 h with BMP9 stimulated group; ^Δ^P < 0.05 and ^ΔΔ^P < 0.01 versus 5 mM metformin for 24 h or 10 mM metformin for 4 h with BMP9 stimulated group; ^ɸɸ^P < 0.01 versus 10 mM metformin for 8 h with BMP9 treated group.

As mentioned above, metformin is an important activator of AMPK, and plays its role via AMPK pathway in most cases. To explore the mechanism that metformin inhibited BMP9-induced HFL-1 cells activation, we investigated the impact of metformin on the expressions of BMP9 signaling key molecules and AMPK. On the one hand, we examine the protein levels of ALK1, p-Smad1/5, t-Smad1/5, AMPK and p-AMPK in HFL-1 cells treated with metformin (0, 1, 5 and10 nM) for 24 h and BMP9 (10 ng/ml) for 0.5 h using western blot assay. On the other hand, western blot assay was also used to detect their protein expressions in HFL-1 cells handled with 10 mM metformin for 2, 4,8 and 24 h and BMP9 (10 ng/ml) for 0.5 h. Cells with Only DMEM were served as control group. As shown in Figures 2A,B, with increasing concentrations of metformin, the expression of p-AMPK was elevated, whereas increased ALK1 expression and phosphorylated Smad1/5 stimulated by BMP9 gradually reduced. For 10 mM metformin, it up-regulated the expression of p-AMPK, but down-regulated the increased expressions of ALK1 and p-Smad1/5 activated by BMP9 in a time-dependent fashion (Figures 2C,D). Thus, it suggests that metformin-restrained proliferation and differentiation of HFL-1 cells might be related to the activation of AMPK pathway and inhibition of ALK1/Smad1/5 signaling pathway.

### Effect of AMPK mutants on BMP9-induced proliferation and differentiation of HFL-1 cells

**FIGURE 3:**
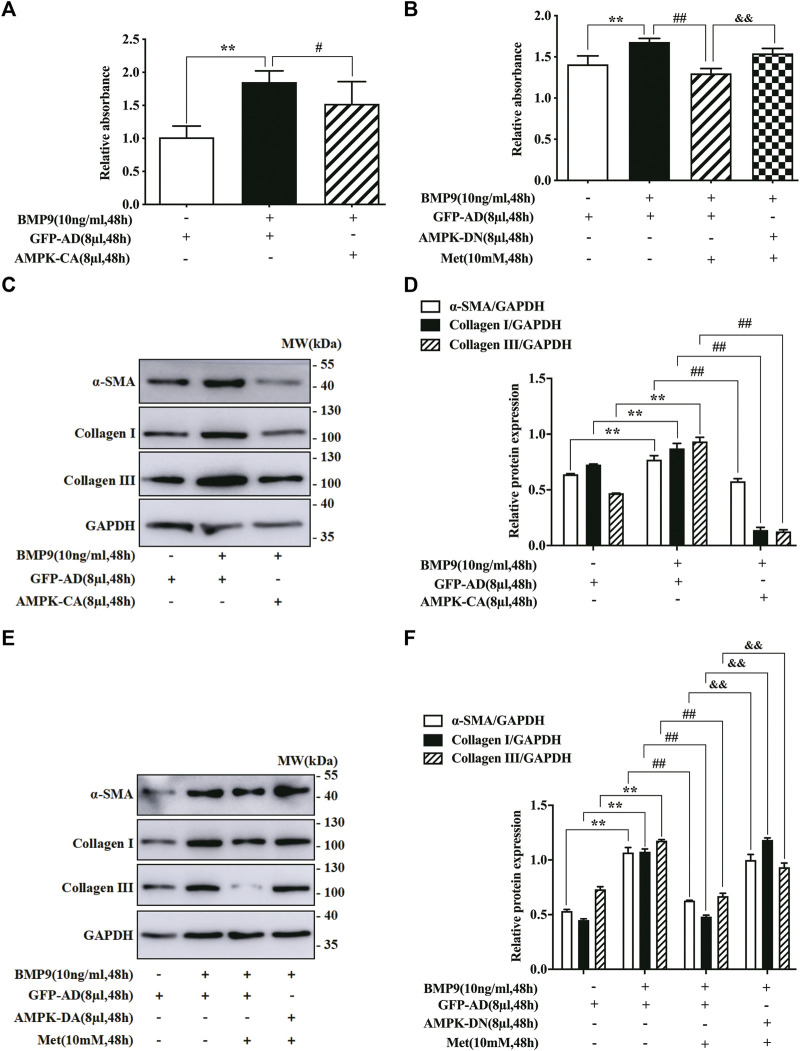
Effect of AMPK mutants on BMP9-induced proliferation and differentiation of HFL-1 cells. **(A)** CCK-8 assay was used to detect the effect of AMPK-CA on the proliferation of HFL-1 cells induced by BMP9 (*n* = 3). **(B)** CCK-8 assay was used to examine the role of AMPK-DN in the inhibitory effect of metformin on BMP9-induced proliferation of HFL-1 cells (*n* = 3). **(C,D)**: The roles of AMPK-CA in the protein expression of fibroblast differentiation markers (ɑ-SMA, Collagen I and Collagen III) were detected by western blot (*n* = 3). **(E,F)**: The effects of AMPK-DN on the protein expression of ɑ-SMA, Collagen I and Collagen III were tested by western blot (*n* = 3). Data are presented as mean ± SD. ^**^ *p* < 0.01 versus only GFP-AD group, ^#^ *p* < 0.05 and ^##^ *p* < 0.01 versus GFP-AD with BMP9 group, ^&&^ *p* < 0.01 versus GFP-AD with BMP9 and metformin group.

In order to verify that the inhibitory effect of metformin on BMP9-mediated response in HFL-1 cells was indeed achieved by activating AMPK pathway, we used the specific AMPK mutants (AMPK-CA and AMPK-DN) to intervene in AMPK activation. For one thing, we examined the effect of AMPK-CA on the proliferation of HFL-1 cells by CCK-8 assay. As shown in Figure 3A, the proliferative abilities of HFL-1 cells in GFP-AD with BMP9 group were higher than those of HFL-1 cells in only GFP-AD group, but the proliferative abilities of HFL-1 cells in AMPK-CA with BMP9 group were lower than those of HFL-1 cells in GFP-AD with BMP9 group. For another thing, we also employed the CCK-8 assay to detect the role of AMPK-DN in the suppressive effect of metformin on BMP9-induced proliferation of HFL-1 cells. Results showed that AMPK-DN reversed the effect of metformin on BMP9-induced HFL-1 cells proliferation, as compared to GFP-AD with BMP9 group (Figure 3B). Meanwhile, we tested the effect of AMPK-CA and AMPK-DN on the protein expressions of fibroblast differentiation markers, including ɑ-SMA, Collagen I and Collagen III, using western blot. Western blot assay indicated that AMPK-CA restrained the up-regulation of these proteins induced by BMP9 in HFL-1 cells **(** Figures 3C,D **)**, while AMPK-DN counteracted the response of metformin to the expressions of ɑ-SMA, Collagen I, and Collagen III in BMP9-treated HFL-1 cells (Figures 3E,F). These results suggest that metformin activates AMPK, thereby inhibiting BMP9-induced proliferation and differentiation of HFL-1 cells.

### Impact of AMPK mutants on the expression of BMP9 downstream signal molecules in HFL-1 cells

**FIGURE 4:**
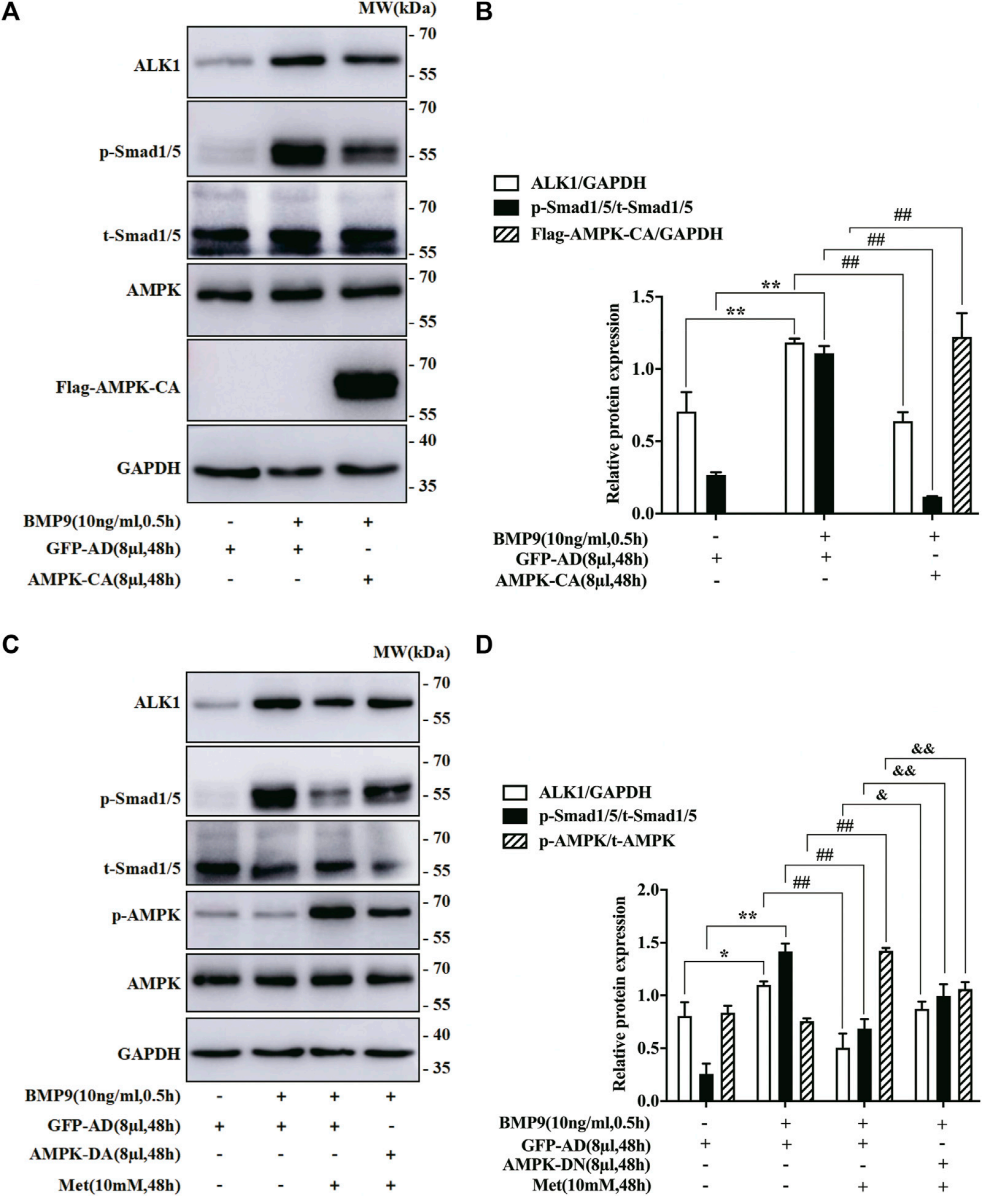
Impact of AMPK Mutants on the expression of BMP9 downstream signal molecules in HFL-1 cells. **(A,B)**: Western blot was employed to examine the effect of AMPK-CA on the expression of ALK1 and p-Smad1/5 (*n* = 3). **(C,D)**: Western blot was applied to detect the effect of AMPK-DN on the expression of ALK1 and p-Smad1/5 (*n* = 3). Data are presented as mean ± SD. ^*^ *p* < 0.05 and ^**^ *p* < 0.01 versus only GFP-AD group; ^##^ *p* < 0.01 versus GFP-AD with BMP9 group; ^&^ *p* < 0.05 and ^&&^ *p* < 0.01 versus GFP-AD with BMP9 and metformin group.

To further ascertain if AMPK activated by metformin suppressed BMP9-induced effects by regulating BMP9 signaling pathway, we employed AMPK-CA and AMPK-DN to infect the HFL-1 cells treated with BMP9, respectively, and then detected the expression levels of BMP9 downstream key molecules (ALK1 and p-Smad1/5) by western blot. As shown in Figures 4A,B, the ability of BMP9 to induce the expressions of ALK1 and p-Smad1/5 was reduced due to the activated response of AMPK-CA to AMPK, but the control vector GFP-AD did not have such response. Furthermore, although metformin effectively inhibited BMP9-induced responses in HFL-1 cells infected with GFP-AD, AMPK-DN infection offset the inhibitory impact of metformin on the up-regulation of ALK1 and p-Smad1/5 by BMP9 (Figures 4C,D). Consequently, these data strongly indicate that the negative effect of metformin on the activation of HFL-1 cells evoked by BMP9 may be achieved by AMPK-regulating ALK1/Smad1/5 cascade.

## Discussion

Pulmonary fibrosis is a common complication with low morbidity and high mortality in chronic lung diseases. Unfortunately, the conventional treatments, such as glucocorticoids combined with immuno-suppressive agents, cannot effectively improve the prognosis of the patients with pulmonary fibrosis (Li et al., 2014; Aso et al., 2019). Two antifibrotic agents (nintedanib and pirfenidone) have been demonstrated their efficacy in phase clinical III since 2015, but they simply slowed the progression of idiopathic pulmonary fibrosis in mild to moderate patients (Collins and Raghu, 2019; Wongkarnjana et al., 2019). Moreover, the cost of nintedanib and pirfenidone is too expensive for ordinary people to afford, each at a cost of almost $100 000 per patient per year (Owens, 2017; Rinciog et al., 2020). Therefore, to clarify its pathogenesis and find more effective and inexpensive drugs is an important issue that needs to be addressed in the treatment of pulmonary fibrosis. In the present study, we employed BMP9 to stimulate HFL-1 cells and found that metformin suppressed BMP9-induced proliferation and differentiation of HFL-1 cells through the activation of AMPK and inhibition of ALK1/Smad1/5 signal.

Metformin, a first-line drug for the clinical treatment of type 2 diabetes, is widely used in metabolic diseases due to its high effectiveness, safety and low cost. Increasing experimental and clinical studies have shown that metformin has multiple health benefits in many disorders (Morales and Morris, 2015; Du et al., 2016; Podhorecka et al., 2017; Soukas et al., 2019; Kang et al., 2020; Torres et al., 2020). For instance, metformin was found to have anti-cancer, anti-aging and anti-oxidant effects. Recently, several studies demonstrated that metformin efficiently alleviated various pulmonary fibrosis, including bleomycin-, radiation- and PM2.5-induced fibrotic models (Choi et al., 2016; Azmoonfar et al., 2018; Farhood et al., 2019; Gao et al., 2020; Xiao et al., 2020). *In vitro* experiments found that metformin inhibited TGF-*β*, especially TGF-*β*1, induced fibroblast activation (Wang et al., 2020). Thus, these findings indicate that metformin might be also an important medication against fibrosis. Moreover, the existing research found that metformin exhibited pleiotropic mechanisms for alleviating lung fibrosis, such as NOX4 inhibition (Jarman et al., 2014; Sato et al., 2016), IGF-1 suppression (Xiao et al., 2020), and mTOR inactivation (Shao et al., 2015; Rangarajan et al., 2018), and so on. In this study, our aims were to determine whether metformin exerted its effect on the activation of HFL-1 by regulating AMPK and BMP9 signaling. As we expected, metformin inhibited BMP9-induced proliferation and differentiation via AMPK activation in HFL-1 cells. Furthermore, this finding was verified by the specific AMPK mutants (AMPK-CA and AMPK-DN). It suggests that metformin activates AMPK, and then restrains BMP9-mediated proliferation and differentiation of HFL-1 cells.

The activation of fibroblasts has been considered as a crucial mechanism that drives the development of pulmonary fibrosis. And a member of transforming growth factor-beta (TGF-*β*) superfamily, TGF-*β*1, has been extensively studied to stimulate fibroblast activation (Li et al., 2015; Wang et al., 2020; Guan et al., 2022; Liu et al., 2022). Like TGF-*β*1, BMP9, another member of TGF-*β* superfamily, binds to two types of transmembrane kinase receptors, type I BMPs (including ALK1and ALK2) and type II BMPs (including BMP receptor type II or activin receptor type II B), forming ligand-receptor complexes with kinase activity. And then regulate gene transcription and expression through smad-dependent and independent pathways, thereby exerting a variety of biological roles (Brown et al., 2005; Asma et al., 2020). Recently research has also found abnormality of BMP9 signaling has closely relationship with fibroblast activation and fibrotic diseases. As an example, aberrant BMP9 signaling has been reported to induce the activation of hepatic stellate cells and promote the progression of liver fibrosis (Breitkopf-Heinlein et al., 2017; Li et al., 2018), but to hinder cardiac fibroblast activation and delay cardiac remodeling and myocardial fibrosis (Morine et al., 2017; Morine et al., 2018), indicating that the response of BMP9 to fibroblast activation varies with cell types. In our study, we demonstrated that BMP9 promoted the proliferation and up-regulated the expression of differentiation markers *α*-SMA, collagen I and collagen III in HFL-1 cells, indicating BMP9 is an effective inducer for the activation of human fetal lung fibroblasts. So far, ALK1 has been regarded as the highest affinity receptor of BMP9 ligand in most types of cells. In most circumstances, BMP9 primarily binds to its receptor ALK1, and then phosphorylates the downstream transcriptional factor Smad1/5. A variety of studies reported that Smad1/5 was activated in fibrotic diseases (Morris et al., 2011; Muñoz-Félix et al., 2016). During hepatic fibrosis, for instance, ligand BMP9 induced the expression of inhibitor of differentiation one *via* the Smad1 pathway, thereby triggering hepatic stellate cells to differentiate into myofibroblasts (Muñoz-Félix et al., 2016). In scleroderma fibroblast, ALK1/Smad1/5 was reported to promote the production of extracellular matrix proteins such as collagen I and connective tissue growth factor (Morris et al., 2011). Consistent with these studies, our research indicated that BMP9 increased the expressions of ALK1 and p-Smad1/5, thereby activated HFL-1 cells.

A couple of evidence has shown that AMPK counter-regulates the TGF-β and BMP signaling pathway in several biological events. As an example, AMPK was reported to inhibit Smad2/3 activity, cell migration and epithelial-mesenchymal transition induced by TGF-β (Lin et al., 2015). Besides, one investigation confirmed that metformin suppressed the osteogenic differentiation induced by BMP6 *via* ALK2/Smad1/5 signaling (Lin et al., 2017), while another investigation found that metformin inhibited BMP9-induced tube formation of HUVECs via ALK1/Smad1/5 pathway (Ying et al., 2017). Additionally, adiponectin regulated the proliferation of pulmonary artery smooth muscle cells through AMPK-regulated BMP/Smad signal pathway (Luo et al., 2021). These results remind us that whether AMPK inhibits the TGF-β/Smad or BMP/Smad-mediated response is a general mechanism. Hence, we explored the effect of AMPK on the BMP9-mediated HLF-1 cells activation in our study. Similar to the above studies, our results uncovered that an active mutant of AMPK (AMPK-CA) imitated the effect of metformin and reduced the expressions of ALK1 and p-Smad1/5 evoked by BMP9, whereas this effect of metformin were rescued by the dominant negative mutant of AMPK (AMPK-DN). Thus, the present study indicates that metformin suppresses BMP9-induced proliferation and differentiation of HFL-1 cells through activating AMPK and hindering ALK1/Smad1/5 signalling, which provides a novel explanation for anti-fibrotic mechanism of metformin.

In summary, this study shows for the first time that metformin can inhibit the proliferation and differentiation of HFL-1 cells induced by BMP9. Furthermore, the present study confirms that metformin counteracts ALK1/Smad1/5 signal pathway to regulate its inhibitory effect on activation of HFL-1 cells *via* activating AMPK. Therefore, our present study delineates the effect of metformin on the activation of lung fibroblasts and the relationship between AMPK and BMP9 signal during this process, strongly supporting the notion that metformin might serve as a preventive and therapeutic agent for lung fibrosis. And also, this study may reinforce our understanding of the anti-fibrotic mechanism of metformin, and provides more theoretical and experimental basis for the clinical application of metformin in the treatment of pulmonary fibrosis. To be sure, there are several limitations in the present study. For instance, the study just focused on *in vitro* experiment to explore the function of metformin on the activation of HFL-1 cells and its mechanism. Accordingly, we should further validate the above results in other cell lines, primary cultured cells, *in vivo* or clinical samples. In addition, we should continue to verify the regulation of AMPK to ALK1 by interfering ALK1 expression.

## Data Availability

The original contributions presented in the study are included in the article/supplementary material, further inquiries can be directed to the corresponding author.
